# Enhancing the performance of pure organic room-temperature phosphorescent luminophores

**DOI:** 10.1038/s41467-019-10033-2

**Published:** 2019-05-08

**Authors:** Kenry, Chengjian Chen, Bin Liu

**Affiliations:** Department of Chemical and Biomolecular Engineering, National University of Singapore, 4 Engineering Drive 4, Singapore, 117585 Singapore

**Keywords:** Optical materials, Nanoscience and technology, Optical materials and structures

## Abstract

Once considered the exclusive property of metal complexes, the phenomenon of room-temperature phosphorescence (RTP) has been increasingly realized in pure organic luminophores recently. Using precise molecular design and synthetic approaches to modulate their weak spin–orbit coupling, highly active triplet excitons, and ultrafast deactivation, organic luminophores can be endowed with long-lived and bright RTP characteristics. This has sparked intense explorations into organic luminophores with enhanced RTP features for different applications. This Review discusses the fundamental mechanism of RTP in pure organic luminophores, followed by design principles, enhancement strategies, and formulation methods to achieve highly phosphorescent and long-lived organic RTP luminophores even in aqueous media. The current challenges and future directions of this field are also discussed in the summary and outlook.

## Abstract (web summary)

Pure organic molecules displaying room-temperature phosphorescence (RTP) are a rapidly emerging class of luminophores. In this Review, the authors discuss the principles for their rational design and development, from the underlying photophysical mechanisms of organic RTP, to enhancement and processing strategies for their practical application.

## Introduction

The photophysical relaxation processes of excited molecules after light absorption, including radiative emission, vibrational relaxation, internal conversion, singlet-triplet intersystem crossing (ISC), triplet state relaxation, energy transfer to ^3^O_2_, etc., form the basis of a wide range of advanced applications, spanning from optoelectronics to photomedicine^1–5^. These relaxation pathways, particularly the ISC between singlet and triplet excited states to yield phosphorescence, have increasingly become the subject of active research and explorations^6–10^. Through a comprehensive understanding and manipulation of the phosphorescence mechanism, novel materials with persistent and stable room-temperature phosphorescence (RTP) have been designed and engineered in the last decade^6–12^.

The phenomenon of RTP has long been regarded as the exclusive feature of metallic and inorganic complexes. The dependence of ISC and phosphorescence lifetime on efficient spin–orbit coupling as well as the prominent effect of oxygen quenching on the triplet excited state in organic luminophores have rendered the realization of pure organic RTP extremely challenging. This explains the rarity of practical and efficient pure organic luminophores as compared to their metal-containing RTP counterparts. Nevertheless, through precise molecular design and synthetic approaches to address directly their weak spin–orbit coupling effect and ultrafast triplet exciton deactivation, pure organic luminophores have been progressively endowed with long-lived and strong RTP in the last several years^6–10^. As compared to metal-containing luminophores, pure organic RTP luminophores, including persulfurated derivatives^13,14^, fluorene derivatives^15,16^, dibenzothiophene and related sulfur-containing heteroaromatic derivatives^17,18^, carbazole and related nitrogen-containing heteroaromatic derivatives^19,20^, sulfur-nitrogen-containing heteroaromatic derivatives^21,22^, borate derivatives^23,24^, and polyacid derivatives^25,26^, etc., are desirable due to the versatility in their molecular design and engineering.

While organic RTP, which was probably originated from a small amount of impurities, was noted as early as 1930s^27^, the observations on RTP of pure organic luminophores have only been reported after another 30–40 years^28–31^. After that, the development of pure organic RTP luminophores has been plagued with uncertainty and a long period of inactivity until recently, when boron difluoride dibenzoylmethane moiety incorporated into poly(lactic acid) (PLA) was observed to display unusual RTP^32^. Since then, a plethora of organic luminophores with long-lived phosphorescence under ambient conditions has been increasingly discovered and realized^6–10^. These notable breakthroughs have sparked active investigations into the rational design and formulation of pure organic luminophores with enhanced RTP performance. Design principles based on halogen bonding^33–35^, H-aggregation^36–38^, and *n*–π transition^39,40^, while RTP enhancement strategies based on co-crystallization^34,41–43^, rigid matrix host–guest system^44–46^, structural modified host–guest system^47,48^, and dopant-based system^49^, have brought about the effective realization of enhanced organic RTP. With their special features and advantages, this class of organic luminophores has been actively demonstrated to have immense potential for specific applications, including organic optoelectronics^50–54^, anti-counterfeiting labeling for advanced data security^36,37,46^, highly sensitive sensing^44,45,55^, and high-contrast time-gated in vitro and in vivo phosphorescence biological imaging^56–60^. Intriguingly, for applications such as optoelectronics and data security, pure organic RTP luminophores could be deposited directly on a wide range of substrates, including semiconducting matrices, plastics, glasses, and papers, and utilized without complicated treatments. For biological applications, however, organic RTP luminophores need to be equipped with sufficient aqueous dispersibility and appropriately processed because the triplet excited state is highly sensitive to oxygen^48^, a molecule that is crucial for physiological functions. As a result, specialized processing strategies are necessary to successfully generate pure organic RTP luminophores with long luminescence lifetime, good water dispersibility, and excellent biocompatibility for biological applications^57–59^. Collectively, this indicates that the effective realization and applications of organic RTP luminophores are an integrated process, covering optical characterization, molecular design, organic synthesis, mechanical study, physical processing, surface modification, and functional evaluation.

Herein, we provide a general overview on the emergence of pure organic RTP luminophores and the rational enhancement of their performance based on structure-property relationship. Specifically, we focus on organic small molecule-based RTP luminophores due to the more established understanding of their RTP mechanisms, as well as the wider explorations into their molecular design, performance enhancement, and potential applications. In contrast, the more complex organic RTP luminophores, including carbon dot- and macromolecule-based (i.e., proteins, starch, carbohydrates, and cellulose) RTP luminophores, will not be discussed in detail as their RTP mechanisms have been less well characterized and understood^61–64^. In this Review, the fundamental mechanism of phosphorescence, which forms the basis of the molecular design of organic luminophores with persistent RTP is first introduced. Specifically, the utilization and manipulation of ISC between singlet and triplet states for realizing stable organic molecular RTP are discussed. By keeping these fundamental principles in mind, we further describe the design principles of pure organic RTP luminophores and the various ingenious strategies used to strengthen and maximize their phosphorescence feature. In particular, we focus on approaches to enhance the ISC rate and lifetime as well as to suppress the non-radiative deactivation pathways of triplet excitons. We next highlight the techniques used to formulate organic RTP luminophores for specific applications. Particular emphasis here is on the nanocrystallization and nanoencapsulation methods used to process organic RTP luminophores for phosphorescence applications in aqueous media. This Review eventually summarizes and presents our perspectives on the current challenges and potential opportunities in the field.

## Mechanism of organic room-temperature phosphorescence

Organic photoluminescent materials have generated considerable interests in recent years due to their tunable luminescent properties, which can be obtained through precise design of molecular structures and control of solid-state intermolecular interactions. When an organic molecule absorbs incident photons, electrons will be excited from ground state to excited state and holes will be simultaneously generated. The generated electron-hole pairs, when they are attracted to each other through electrostatic force, are commonly known as excitons.

**Fig. 1:**
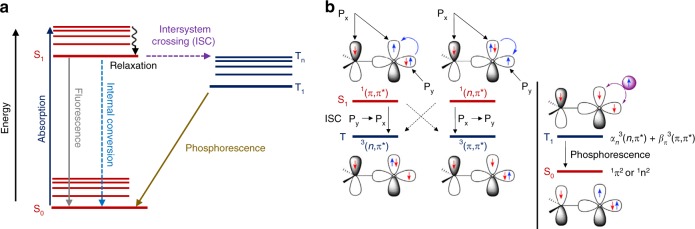
Fundamental mechanism of organic room-temperature phosphorescence (RTP) phenomenon. **a** Jablonski diagram illustrating the different photophysical relaxation processes, particularly the intersystem crossing (ISC) between singlet and triplet states, which forms the basis for phosphorescence of organic luminophores. **b** Schematic illustration of the El-Sayed’s rule for ISC and its utilization for controlling phosphorescence decay rate based on the molecular-orbital hybridization of the lowest triplet states. **b** is adapted with permission from ref. ^39^. Copyright (2016) Cell Press

In general, the emission characteristics of organic photoluminescent molecules are significantly dependent on the electronic transitions between the different states of excitons. By controlling the properties of excitons, such as their configuration, energy level, and lifetime, the emission properties of organic luminescent molecules can be fine-tuned. The numerous possible photophysical pathways taking place between the singlet and triplet excited states in organic molecules are summarized in the Jablonski diagram below (Fig. 1a).

As one of the two radiative photophysical relaxation processes, phosphorescence occurs when there is an ISC from an excited energy state (usually a singlet state) to another state with a higher spin multiplicity (usually a triplet state), followed by radiative deactivation to the ground state. In contrast to fluorescence, phosphorescence is inherently slower and possesses a longer lifetime. To yield phosphorescence, it is imperative to have an effective non-radiative ISC between the isoenergetic singlet excited state (S_1_) and T_n_. The rate of ISC is an intrinsic feature of organic molecule and therefore, dependent on its electronic configuration and electronic level. To populate T_n_ from S_1_, an efficient spin–orbit coupling is essential.

In accordance to El-Sayed’s rule, effective spin–orbit coupling and a high ISC rate (*k*_ISC_) can be achieved in pure organic luminophores if the non-radiative transition from singlet to triplet excited states involves different molecular-orbital configurations because of the effective overlapping orbitals (Fig. 1b)^39,65^. For instance, ISC can occur from ^1^(*n*,π*) singlet to^3^(π,π*) triplet excited states or from ^1^(π,π*) singlet to ^3^(*n*,π*) triplet excited states. However, ISC from singlet to triplet excited states with similar electronic configurations, such as from ^1^(*n*,π*) to ^3^(*n*,π*) or from ^1^(π,π*) to ^3^(π,π*), is not favorable due to minimal orbital overlapping, which results in inefficient spin–orbit coupling. Consequently, the presence of *n* orbitals perpendicular to π orbitals is beneficial to facilitate a strong spin–orbit coupling and promote the ISC from singlet to triplet excited states. Furthermore, the electronic configuration of triplet excited state is a combination of two configurations with varying percentages of *α*_*n*_^3^(*n*,π*) + *β*_π_^3^(π,π*), in which *α*_*n*_ + *β*_π_ = 1, and because of this, the rate of phosphorescence relaxation becomes tunable. As a result, the molecular design and precise tuning of the triplet excited state with appropriate proportions of hybrid (*n*,π*) and (π,π*) configurations are important to achieve effective long-lived RTP luminophores^39^.

In addition to mixing singlet and triplet states with different electronic configurations to enhance spin–orbit coupling, the rate of the ISC process from S_1_ to T_n_ can be considerably strengthened by introducing a small splitting energy gap (Δ*E*_ST_) between these excited states^39^. Separately, the inclusion of heavy-metal atoms, such as platinum, iridium, and halogen atoms (e.g., bromine and iodine atoms), is also beneficial in triggering spin–orbit coupling to strengthen the rate of ISC from S_1_ to T_n_, as well as the rate of radiative relaxation from T_1_ to S_0_^33,34,56^. This is necessary for phosphorescence to compete with other photophysical processes, such as fluorescence, internal conversion, non-radiative deactivation pathways from T_1_ to S_0_, as well as oxygen quenching under ambient conditions. In fact, luminophores with high ISC quantum efficiency typically display fast *k*_ISC_, which can compete with fluorescence decay and internal conversion in the S_1_ depopulation process. Because of this, another crucial requirement for RTP to occur in pure organic luminophores is the higher rate of radiative transition (*k*_P_) than that of non-radiative transition from the triplet excited state to ground state (*k*_nr_). Under ambient conditions, the non-radiative loss typically surpasses the radiative decay^66,67^. At the same time, since ISC is a forbidden process according to the principle of angular momentum conservation, it takes place on a longer time scale and triplet exciton luminescence has longer lifetimes. With the increase in luminescence lifetime of triplet excitons, they are highly susceptible to external environmental factors, including heat and oxygen in the air^68^. Consequently, it is extremely challenging to realize bright and highly efficient luminescence from long-lived triplet excitons under ambient conditions, especially in pure organic luminophores. The suppression of non-radiative relaxations and external quenching thus becomes a crucial factor in realizing highly effective and bright RTP in pure organic luminophores^48,67^.

To display persistent RTP, pure organic luminophores also need to have a slow T_1_ decay rate. One of the most promising ways to achieve this is to stabilize the lowest triplet excited states T_1_ by forming numerous lower-lying energy states with suppressed non-radiative decay in H-aggregates^36^. With the increase in the number of such triplet excited states at lower-energy level (T_1_*), the decay rate from T_1_ to S_0_ decreases considerably. This is because the newly formed triplet excited states can function as emission-forbidden energy trapping states, which will eventually lead to the generation of long-lived persistent organic RTP.

Altogether, to achieve pure organic RTP luminophores with both high phosphorescence quantum yield and persistent phosphorescence lifetime, several factors need to be fulfilled. These include: (1) an efficient spin flipping due to the ISC process from S_1_ to T_n_, (2) a low non-radiative relaxation rate to promote phosphorescence from T_1_ to S_0_, and (3) a slow decay of T_1_ to realize long-lived phosphorescence. Various rational design principles and enhancement strategies have thus been geared towards meeting these requirements recently, which are discussed in the following sections.

## Design of pure organic room-temperature phosphorescent luminophores

**Fig. 2:**
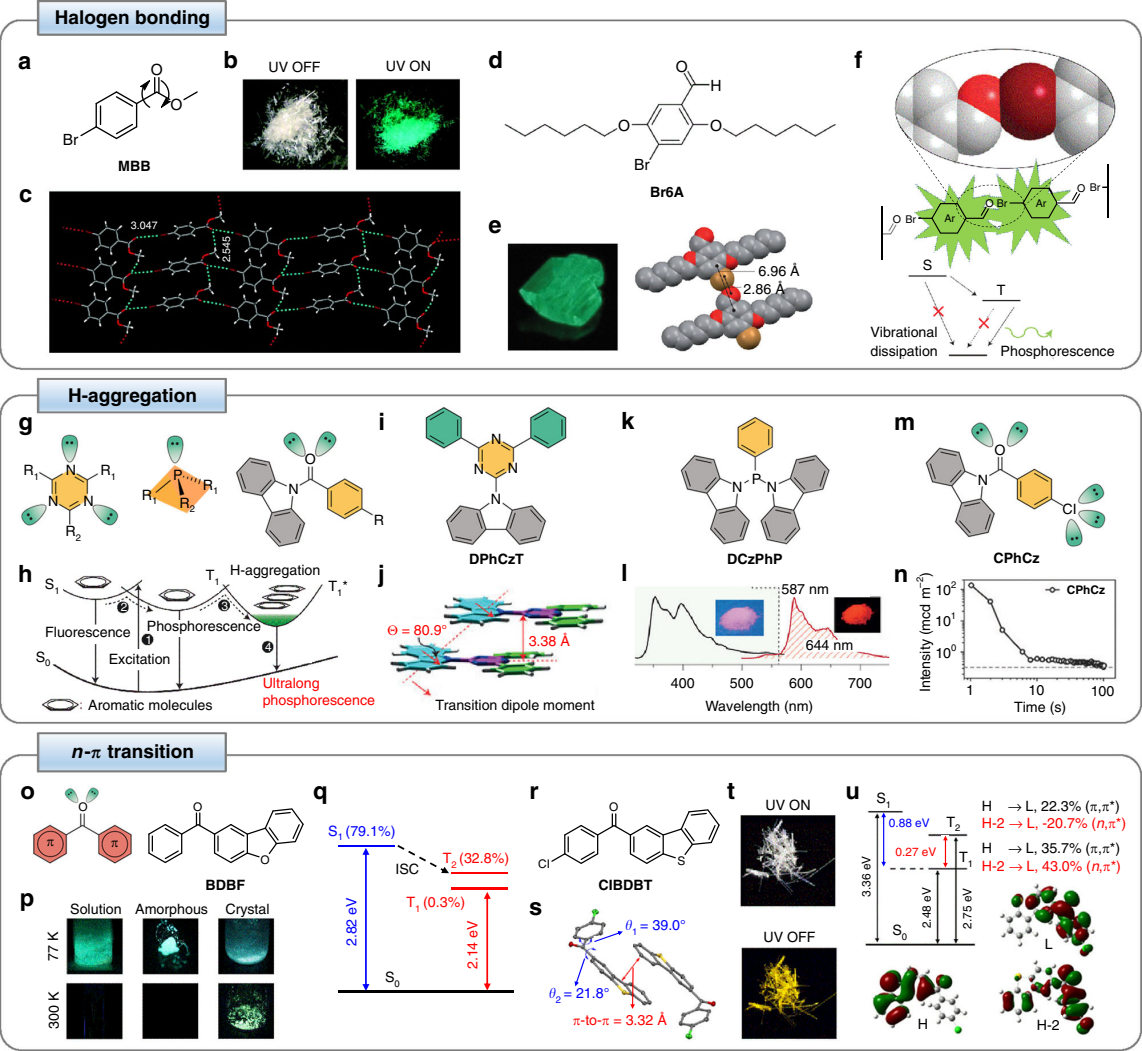
Major design strategies in realizing pure organic RTP based on halogen bonding, H-aggregation, and *n*–π transition. **a** Chemical structure of **MBB**. **b** Photographs of the different states of **MBB** crystals under normal light (left) and UV light (right) illuminations at ambient condition. **c** Molecular packing structure of **MBB** crystal. **d** Chemical structure of luminophore **Br6A**. **e** Photograph of **Br6A** crystal under UV light irradiation (left) and the molecular packing structure of **Br6A** crystal, illustrating the halogen bond between carbonyl oxygen and bromine (right). **f** Schematic illustration of the realization of phosphorescence of **Br6A** in crystal form. **g** General chemical structures of different organic molecules designed to control the lifetime of excited states through the incorporation of alkyl or aromatic substituents to facilitate H-aggregate formation and stabilize triplet exciton. R_1_ and R_2_ can be H, Cl, C_2_H_5_, C_12_H_8_N, etc. **h** Schematic illustration of the generation of lower-lying energy states for stabilization of the lowest triplet excited state (T_1_) to achieve long organic phosphorescence. **i** Chemical structure of **DPhCzT**. **j** Crystal structure of **DPhCzT** depicting the H-aggregate formation through the measured angle of 80.9^o^ between the interconnected axis and transition dipoles. **k** Chemical structure of **DCzPhP**. **l** Steady-state photoluminescence (left) and phosphorescence (right) profiles of **DCzPhP**. Insets illustrate the different states of **DCzPhP** before (left) and after (right) switching off the 365-nm excitation under room temperature. **m** Chemical structure of **CPhCz**. **n** Luminescence decay of **CPhCz** after a 5-min visible light excitation. **o** Chemical structures of rationally designed organic molecule with carbonyl group and non-bonding electrons (left) and **BDBF** (right). **p** Photographs of the different states of **BDBF** in solution, amorphous, and crystal forms at 77 and 300 K. **q** Calculated energy levels with the proportion of *α*_*n*_ of the different excited states of **BDBF**. **r** Chemical structure of **CIBDBT**. **s** Crystal structure of **CIBDBT**. **t** Photographs of the different states of **CIBDBT** before (top) and after (bottom) switching off the 365-nm excitation source. **u** Calculated energy levels, electronic transitions, and frontier molecular orbitals of the highest occupied molecular orbitals H, H-2, and the lowest unoccupied molecular orbital L. **a**, **b**, and **c** are adapted/reproduced with permission from ref. ^33^. Copyright (2010) American Chemical Society. **d**, **e**, and **f** are adapted/reproduced with permission from ref. ^34^. Copyright (2011) Springer Nature. **g** is adapted with permission from ref. ^36,37^. Copyright (2015) Springer Nature and (2017) Wiley-VCH Verlag GmbH & Co. **h**, **i**, **j**, **k**, and **l** are adapted/reproduced with permission from ref. ^36^. Copyright (2015) Springer Nature. **m** and **n** are adapted/reproduced with permission from ref. ^37^. Copyright (2017) Wiley-VCH Verlag GmbH & Co. **o**, **p**, and **q** are adapted with permission from ref. ^39^. Copyright (2016) Cell Press. **r**, **s**, **t**, and **u** are adapted/reproduced with permission from ref. ^40^. Copyright (2017) Springer Nature

Tremendous efforts have been channeled towards formulating rational rules for designing bright and long-lived pure organic RTP luminophores in recent years. In general, this unique class of organic luminophores can be designed systematically through major strategies based on halogen bonding^33–35^, H-aggregation^36–38^, and *n*–π transition^39,40^ (Fig. 2). Collectively, these strategies aim to strengthen the ISC transitions from the singlet to triplet excited states and simultaneously mitigate the prominent non-radiative relaxations of triplet excited states.

One of the earliest attempts in realizing persistent organic RTP luminophores relied on the crystallization-induced restriction of intramolecular motions (Fig. 2a–c)^33^. In this study, the crystals from a series of organic luminophores based on benzophenone and its halogenated derivatives, as well as methyl 4-bromobenzoate (**MBB**) were prepared (Fig. 2a). All luminogens were generally non-emissive when they existed in the solution form due to annihilation of triplet excitons by active intramolecular rotations and vibrations. However, in the crystalline state, both crystal lattices and intermolecular interactions confined the intramolecular motions, resulting in bright RTP under ultraviolet (UV) irradiation at room temperature (Fig. 2b). While this study has largely focused on the effect of crystallization, considerable attention has also been paid to the role of halogen bonding, which was one of the intermolecular interactions central to RTP emission of organic crystals. Halogen bonding is generally a weak non-covalent interaction between a nucleophilic atom or a negatively charged ion and a positively polarized halogen atom. Owing to its directionality, this type of bonding has become increasingly crucial in crystal engineering. Heavy halogen atoms, such as bromine and iodine, are able to introduce heavy atom effect when bonded with luminophores to strengthen the efficiency of ISC process between singlet and triplet states. A closer look at the molecular packing of the crystal structure of **MBB** revealed the locking and stabilization of its conformation by numerous C–H**···**O and C=O**···**Br–C intermolecular interactions and halogen bonds, with distances of 2.55 and 3.05 Å, respectively (Fig. 2c). This illustrates the crucial roles of carbonyl group (C=O) and heavy atom of organic luminophores in rigidifying their structure to achieve more efficient spin–orbit coupling and enable bright phosphorescence.

The more pronounced effect of halogen bonding in facilitating a directed heavy atom effect to generate organic RTP was reported around the same time (Fig. 2d–f)^34^. Here, a small molecule of 2,5-dihexyloxy-4-bromobenzaldehyde (**Br6A**) was firstly synthesized (Fig. 2d). When **Br6A** luminophores were in solution, they did not display any halogen bonding. Under this condition, there was inefficient generation of triplet excitons, resulting in ineffective RTP. This was evident from the weak fluorescence of **Br6A** in chloroform, with a lifetime of 0.5 ns and a quantum yield of 0.5%, when it was excited at 360 nm. However, **Br6A** crystals displayed distinct green emission with a phosphorescent lifetime of 5.4 ms and a quantum yield of 2.9% (Fig. 2e). As revealed through single-crystal X-ray diffraction (XRD), the molecular packing structure of **Br6A** crystal revealed the close proximity of carbonyl oxygen with bromine within the crystal. In fact, the C=O**···**Br angle of 126^o^ strongly indicates the presence of halogen bond. Furthermore, the distance of 2.86 Å between the carbonyl oxygen and bromine atoms is one of the shortest distances among the halogen bonds. The crystallization-induced halogen bond was most likely the reason for the strong phosphorescence detected from the crystalline **Br6A**, although heavy atom effect would also play a role (Fig. 2f). More clearly, the strong halogen bonding of **Br6A** crystal induced a unique directed heavy atom effect specifically at the triplet generation sites, which could significantly boost triplet generation, activate triplet emission, and suppress fluorescence and vibrational loss to enhance phosphorescence. It was found that **Br6A** suffered from self-quenching and therefore, its RTP was extremely weak and short-lived. Encouragingly, numerous studies have demonstrated that the phosphorescence quantum efficiency and lifetime of **Br6A** could be significantly enhanced through various ingenious RTP enhancement strategies^34,41,44^.

In addition to halogen bonding, another increasingly explored strategy to design organic RTP luminophores with long lifetime is the stabilization of triplet excitons through molecular H-aggregation (Fig. 2g–n)^36,37^. In one of the more recent studies, a series of carbazole-based luminophores consisting of N, O, and P atoms was prepared (Fig. 2g). These atoms are able to expedite the spin-forbidden ISC of singlet to triplet excited states^69^. Furthermore, planar configurations with well-defined aromatic substituents were incorporated in the molecular design to stimulate the parallel alignment of the molecules and facilitate the generation of stable H-aggregates, which could possibly enhance the singlet excited state lifetimes^70^. The unique design features demonstrated in this work were largely inspired by the generation of persistent RTP in inorganic luminophores based on charge carrier trapping^71^. It was hypothesized that the existence of an energy trapping state T_1_* in these organic luminophores could lead to stabilization of triplet excited states for the generation of long-lived organic RTP (Fig. 2h). To test this idea, 4,6-diphenyl-2-carbazolyl-1,3,5-triazine (**DPhCzT**) was synthesized (Fig. 2i) to show green RTP with a considerably long phosphorescence lifetime of 1.06 s and a quantum efficiency of 1.25%. First-principle time-dependent density functional theory (TD-DFT) revealed a small energy gap of < 0.3 eV between the lowest singlet and triplet excited states in **DPhCzT** monomer, which favored the singlet-to-triplet ISC process^72^. Moreover, XRD analysis of the single-crystal structure of **DPhCzT** uncovered the presence of H-aggregates as the measured angle between the transition dipoles and the interconnected axis was 80.9^o^, which was larger than the critical value of 54.7^o^ differentiating J-aggregation and H-aggregation^36^ (Fig. 2j). To demonstrate the general applicability of triplet exciton stabilization through molecular H-aggregation strategy in inducing persistent organic RTP, other pure organic luminophores with N, O, and P atoms, including di(9*H*-carbazolyl)-phenylphosphine (**DCzPhP**), were synthesized (Fig. 2k). By tailoring its molecular structure, **DCzPhP** was able to emit bright red RTP (Fig. 2l).

Extending the design concept of triplet exciton stabilization through H-aggregation, a range of organic RTP luminophores with halogen substituents, including (9*H*-carbazol-9-yl)(4-chlorophenyl)-methanone (**CPhCz**), was synthesized in a more recent study (Fig. 2m)^37^. Owing to the synergistic integration of two functional strategies, these aromatic amide derivatives could be excited by visible light to emit organic RTP. More clearly, strong intermolecular interactions on the xy plane redshifted the absorption of the luminophores, while on the *z*-axis, molecules with H-aggregations stabilized the triplet excitons of the long-lived organic RTP. **CPhCz** had a high phosphorescence efficiency of 8.3% and displayed a long phosphorescence duration of 104 s under a brightness identifiable by naked eye (i.e., 0.32 mcd m^-2^) (Fig. 2n).

Stabilization of triplet excitons for long-lived organic RTP can also be achieved by controlling the intermolecular interactions in the crystalline state using external stimuli^73^. In fact, several recent studies have demonstrated the realization of ultralong organic RTP through UV photoactivation-enabled manipulation of intermolecular interactions^60,74^. For example, in one of the studies, the phenomenon of photoinduced RTP was realized in 10-phenyl-10*H*-phenothiazine-5,5-dioxide derivatives through UV light-enhanced π–π interactions of aromatic rings^60,74^. In another work, photoactivated dynamic RTP was achieved in a series of organic luminophores with a triazine core, a carbozole unit, and alkoxyl chains with different length^73^. By irradiation under UV light for 8 min, the organic luminophores could be activated to exhibit RTP with emission lifetimes increasing from 1.8 to 1330 ms. These luminophores could be deactivated to emit short-lived phosphorescence by leaving them under ambient conditions for about 3 h or thermally treating them. Separately, instead of attaching alkyl chains onto the triazine core to fine-tune the molecular configuration, the flexible alkyl chains can be introduced to link carbazole moieties with heavy atoms to modulate spin–orbit coupling and strengthen intermolecular heavy atom effect for persistent organic RTP^75^. Carbazole-based organic RTP luminophores could be designed to exhibit yellow persistent RTP emission with a high quantum yield of 39.5% and a long lifetime of about 200 ms^75^. Interestingly, although the strong persistent RTP emission was initially attributed to the carbazole derivatives, additional purification of the same luminophores revealed the disappearance of the yellow RTP^76^. As such, the small traces of impurities existed in the organic compounds were most likely responsible for the observed persistent RTP.

Another major strategy to promote singlet-triplet ISC in order to populate triplet states and achieve long-lived organic RTP is the enhancement of spin–orbit coupling based on *n*–π transition (Fig. 2o–u)^39,40^. This design concept was well documented in a recent structure-property relationship study, which investigated the effect of molecular orbital on the phosphorescence wavelength, lifetime, and efficiency^39^. Here, a series of benzophenone-based luminophores integrating carbonyl groups with non-binding electrons and different π-conjugated groups was prepared (Fig. 2o). Taking 1-(dibenzo[*b*,*d*]furan-2-yl)phenylmethanone (**BDBF**) as an example, carbonyl groups were incorporated to provide *n* orbitals, which could enhance spin–orbit coupling to trigger ISC from the excited singlet state S_1_ to different triplet states T_n_. Meanwhile, π-conjugated units were introduced to provide π orbitals and endow T_1_ state with ^3^(π,π*) configuration, which could induce a slow phosphorescence rate and a persistent phosphorescence lifetime. By varying the π-conjugated units, the (*n*,π*) and (π,π*) molecular orbitals could be mixed to generate tunable T_1_ state with distinct ^3^(π,π*) configuration and energy levels, which in turn, enabled the tuning of phosphorescence color, lifetime, and quantum yield. The luminophores prepared in this work were generally not luminescent in both solution and amorphous states, but became highly emissive when they were transformed into crystals at room temperature (Fig. 2p). For example, **BDBF** crystals displayed RTP with a phosphorescence lifetime of 232 ms and efficiency of 34.5%. First-principle TD-DFT calculations were performed on **BDBF** to unravel the mechanism of its RTP (Fig. 2q). It was noted that, because of a high Δ*α*_*n*_ (which is defined as *α*_*n*,S1 _– *α*_*n*,_T_n_) of 46.3% between S_1_ and T_2_ (where *α*_*n*,S1_ and *α*_*n*,T2_ were 79.1% and 32.8%, respectively) indicating an enhanced *n*–π transition and stronger spin–orbit coupling, as well as a high *β*_π,T1_ of 99.7% indicating the prominent ^3^(π,π*) configuration of T_1_, **BDBF** possessed a long phosphorescence lifetime.

Interestingly, the *n*–π transition design concept can also be extended to enable white phosphorescence emission from a single pure organic luminophore at room temperature. Pure organic white RTP was realized through intrasystem mixing of dual-emission bands, which were originated from the high- and low-lying triplet excitons (Fig. 2r–u)^40^. Fundamentally, if there are two radiative relaxations from both the higher and lower triplet excited states, dual phosphorescence could be observed^77,78^. In this study, four arylphenone derivatives consisting of heavy halogen atom (i.e., Br, Cl, or F), carbonyl group, and π-extended dibenzothiophene subunit, particularly 4-chlorobenzoyldibenzothiophene (**CIBDBT**), were prepared (Fig. 2r). While these organic molecules were not luminescent in solutions, they were capable of displaying dual RTP emission in crystalline state. The mechanism that enables this unique phenomenon is the combined effect of: (1) enhanced ISC due to the presence of lone pair electrons in carbonyl group and heavy halogen atom, with (2) multiple triplet excited states having distinct energy levels and orbital configurations due to the π-extended dibenzothiophene subunit. Intriguingly, upon 365 nm UV irradiation, **CIBDBT** could emit white phosphorescence, which turned yellow after the removal of excitation source (Fig. 2t). The crystal structure of **CIBDBT** showed that the luminophore existed in dimeric units linked by π–π interaction (Fig. 2s). The small dihedral angle of 21.8^o^ between dibenzothiophene and the carbonyl group indicates their strong conjugation within the luminophore. This raises the possibility that (*n*,π*) from the carbonyl group and (π,π*) could synergistically form hybrid triplet excited states. In fact, theoretical calculations showed that there were two low-lying triplet excited states (i.e., T_1_ and T_2_) below the lowest excited singlet state (i.e., S_1_), where the lower-energy T_1_ and the higher-energy T_2_ are the mixed states dominated by (π,π*) and (*n*,π*) transitions, respectively (Fig. 2u). The (π,π*) transition localized to the benzophenone unit (i.e., the lowest unoccupied molecular orbital L) from the π-conjugated dibenzothiophene (e.g., the highest occupied molecular orbital H), while the (*n*,π*) transition exhibited charge transfer from deeper orbitals (e.g., H-2) to L. All these suggest the importance of the existence of both low- and high-lying triplet excited states with (π,π*) and (*n*,π*) transitions in the design of organic persistent dual RTP luminophores.

## Enhancement of organic room-temperature phosphorescence

It is essential for pure organic RTP luminophores to be bright and with long lifetime for practical applications. While a majority of the pure organic luminophores display weak and short-lived RTP in the scale of ms, encouragingly, the RTP performance of organic luminophores can be greatly strengthened. To this end, various approaches which are capable of suppressing the non-radiative deactivation pathways and reducing the quenching of triplet excitons, as well as enhancing their ISC rate and lifetime have been increasingly explored. These mainly include co-crystal assembly^34,41–43^, rigid matrix host–guest system^44–46^, structural modified host–guest system^47,48^, and dopant-based system^49^.

### Co-crystallization

**Fig. 3:**
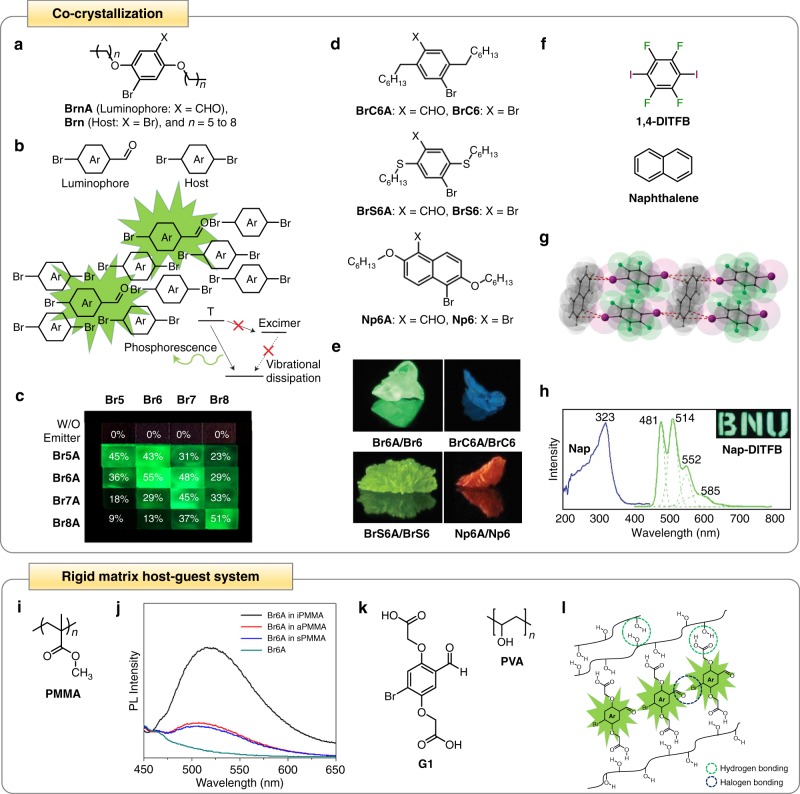
Enhancement of organic RTP based on co-crystallization and rigid matrix host–guest system. **a** Chemical structures of a pair of aldehyde luminophore (**BrnA**) and analogous host (**Brn**) used in co-crystallization, where X are CHO and Br for luminophore and host, respectively, and *N* refers to the alkoxy chain length with varying amount of carbon atoms. **b** Schematic illustration of the mechanism of RTP enhancement based on co-crystallization, where luminophores (i.e., brominated aromatic aldehydes) are substituted into the host crystals (i.e., dibrominated analogs to the luminophores) and isolated to minimize the excimer-induced self-quenching and enhance the overall RTP quantum yield. **c** Photographs of the co-crystals formed from **BrnA** (i.e., **Br5A**, **Br6A**, **Br7A**, and **Br8A**) and **Brn** (i.e., **Br5**, **Br6**, **Br7**, and **Br8**) with different alkoxy chain lengths consisting of five to eight carbon atoms, under 365 nm UV light excitation. The numerical values within the co-crystals indicate their photoluminescence quantum efficiencies. **d** Chemical structures of different luminophore/host pairs, i.e., **BrC6A/BrC6**, **BrS6A/BrS6**, and **Np6A/Np6**. **e** Photographs of the co-crystals mixed from **Br6A/Br6**, **BrC6A/BrC6**, **BrS6A/BrS6**, and **Np6A/Np6** combinations under 365-nm UV light irradiation. **f** Chemical structures of **1,4-DITFB** and Naphthalene. **g** Packing structure of the co-crystal of **1,4-DITFB** and Naphthalene (Nap-**DITFB**). **h** Excitation (in blue) and emission (in green) profiles of Nap-**DITFB** co-crystal. Inset shows the co-crystal of **1,4-DITFB** and Naphthalene under 365-nm UV light irradiation. **i** Chemical structure of the host PMMA. **j** Photoluminescence profiles of pure **Br6A** luminophore and **Br6A** embedded in different variants of PMMA. **k** Chemical structures of the guest **G1** and the host PVA. **l** Schematic illustration of the mechanism of RTP enhancement based on rigid matrix host–guest system, where **G1** is embedded within PVA and the PVA–PVA hydrogen bonds, the **G1**-PVA hydrogen bonds, as well as the **G1**–**G1** halogen bonds collectively contribute to organic RTP enhancement. **a** and **c** are adapted/reproduced with permission from ref. ^41^. Copyright (2014) American Chemical Society. **b**, **d**, and **e** are adapted with permission from ref. ^34^. Copyright (2011) Springer Nature. **f** and **h** are adapted with permission from ref. ^42^. Copyright (2012) The Royal Society of Chemistry. **g** is reproduced with permission from ref. ^6^. Copyright (2015) The Royal Society of Chemistry. **i** and **j** are reproduced with permission from ref. ^44^. Copyright (2013) American Chemical Society. **k** and **l** are adapted/reproduced with permission from ref. ^45^. Copyright (2014) Wiley-VCH Verlag GmbH & Co

Co-crystallization relies on the synergistic co-assembly of luminophores with host crystals to achieve a directed heavy atom effect in order to trigger a more effective ISC process to improve RTP (Fig. 3a–g)^34^. In one of the pioneering reports demonstrating this enhancement concept, a pure organic luminophore **Br6A** was synthesized and co-crystallized with its bi-halogenated analog host of 2,5-dihexyloxy-1,4-dibromobenzene (**Br6**) to generate a co-crystal of **Br6A**/**Br6** (Fig. 3a)^34^. **Br6** has a similar crystal structure and size to **Br6A**. Therefore, the presence of **Br6** in the co-crystals could minimize the formation of excimers and suppress the self-quenching of **Br6A** to yield more effective RTP with a high quantum yield of 55%, almost 20-fold brighter than that of **Br6A** alone (Fig. 3b). It is interesting to note that the emission of **Br6A**/**Br6** co-crystals was still originated from the pure **Br6A** crystals as evidenced by their narrow excitation band, which is within the absorption spectrum of **Br6A** but not **Br6**. XRD analysis of the crystal structures of both **Br6A** and **Br6** revealed their similar packing motifs and aromatic ring distances. This suggests that **Br6A** luminophore was incorporated into the **Br6** host through substitution.

It is important to highlight that, in addition to luminophores and their analogous host crystals of the same sizes, mismatched luminophore-host pairs with different alkoxy chain lengths were also generated, albeit with lower phosphorescence quantum efficiencies. This was reported in a recent study, which explored the emission brightness of a series of co-crystals mixed from different combinations of aldehydes and their analogous host crystals with varying alkoxy chain lengths between five and eight carbon atoms (Fig. 3c)^41^. Significantly brighter emission could only be achieved with matched luminophore-host pairs of similar sizes. In fact, with an increase in the size differences between the luminophores and hosts, a corresponding decrease in quantum efficiencies of the resultant mixed crystals was observed. Separately, by coupling luminophores possessing different electron densities and triplet levels with their analogous host crystals, additional organic co-crystals with different emission colors could be engineered (Fig. 3d, e)^34^. For example, co-crystals constructed from the luminophore/host combinations of 4- bromo-2,5-diheptylbenzaldehyde/1,4-dibromo-2,5-diheptylbenzene (**BrC6A**/**BrC6**), 4-bromo-2,5-bis(hexylthio)benzaldehyde/(2,5-dibromo-1,4-phenylene)bis(hexylsulfane) (**BrS6A**/**BrS6**), and 5-bromo-2,6-bis(hexyloxy)-1-naphthaldehyde/1,5-dibromo-2,6-bis(hexyloxy)naphthalene (**Np6A**/**Np6**) could emit bright blue, yellow, and orange phosphorescence, respectively, under ambient conditions.

In addition to the co-crystallization of luminophores with their analogous host crystals, organic emitters can be paired up with non-analogous host crystals (Fig. 3f–h). In one study reporting such pairing, 1,4-diiodotetrafluorobenzene (**1,4**-**DITFB**) and the polyaromatic hydrocarbon naphthalene (Nap) were prepared and co-crystallized to generate Nap-**DITFB** (Fig. 3f)^42^. Here, **1,4**-**DITFB** and Nap served as halogen bonding donor and π-type halogen bonding acceptor, respectively. While polyaromatic hydrocarbons are typically good luminescent materials, it is challenging to observe their phosphorescence. The incorporation of **1,4-DITFB** as a halogen bonding donor was, therefore, anticipated to improve the phosphorescence of the co-crystals. Analysis on the crystal structure of Nap-**DITFB** revealed the presence of C–I**···**π halogen bonding in the co-crystals (Fig. 3g). The stability of the co-crystal structure was also maintained by additional weak intermolecular interactions, notably C–H**···**I hydrogen bonding, C-F**···**F-C contact, and edge-to-edge π-π stacking. With the maximum excitation at 323 nm, Nap-**DITFB** could emit strong green phosphorescence with a phosphorescence lifetime of 0.067 ms (Fig. 3h). Altogether, the introduction of heavy atom **1,4-DITFB** diluted the solid-state naphthalene concentration to suppress its self-quenching, while the C–I**···**π halogen and C–H**···**I hydrogen interactions rigidified the molecular environment, resulting in an improved ISC process and considerably enhanced organic RTP.

### Rigid matrix host–guest system

Although bright organic RTP can be achieved through co-crystallization based on a directed heavy atom effect, the practical applications of the resultant organic crystalline luminophores are still largely hampered by their crystal quality^79^. For device fabrication and processing applications, including sensors, solid-state lighting, and organic light-emitting diodes, amorphous solids and polymers are much easier to process than crystalline materials. However, amorphous organic systems with bright RTP are challenging to realize. Rigorous vibration and diffusion movements, such as *α* and *β* transitions, are typically rampant in amorphous phase under ambient conditions^45^. These unavoidable phenomena in amorphous matrices favor the vibrational loss of triplet excitons of organic luminophores, causing significant quenching of their phosphorescence^80,81^. As such, suppressing the vibrational and diffusional motions in an amorphous environment is extremely important as these movements compete with phosphorescent decay.

To address this issue, increasing activities have been geared towards confining pure organic RTP luminophores within carefully chosen rigid amorphous polymer matrices with minimal amorphous phase relaxations, such as poly(methyl methacrylate) (PMMA) and polyvinyl alcohol (PVA) (Fig. 3i–l)^44,45^. With this notable feature, these polymer matrices were anticipated to mitigate the vibrational triplet decay to realize emissive triplet relaxation and boost the overall phosphorescence efficiency. Taking the luminophore **Br6A** as an example, it could be embedded within PMMA to improve its phosphorescence brightness and efficiency (Fig. 3i–j)^44^. Specifically, **Br6A** was embedded within a series of PMMA with different tacticity, i.e., atactic PMMA (aPMMA), isotactic PMMA (iPMMA), syndiotactic PMMA (sPMMA) (Fig. 3i) and their RTP emission intensity was evaluated. Among all samples, the iPMMA-embedded **Br6A** showed the brightest phosphorescence, with a phosphorescence quantum efficiency up to 7.5% (Fig. 3j). This was attributed to the high isotacticity and low β-relaxation characteristic of iPMMA. Generally, amorphous PMMA with different tacticity arrangements displays varying degrees of β-relaxation, which influences the vibrational loss of triplet excited states and phosphorescence efficiency. In fact, the degree of β-relaxation of amorphous PMMA decreases with decreasing syndiotacticity, but increases with decreasing isotacticity. Therefore, with increasing isotacticity in iPMMA, its β-relaxation decreased correspondingly and phosphorescence brightness and quantum efficiency of the embedded **Br6A** were significantly improved.

The suppression of both vibrational and diffusional motions in an amorphous environment under ambient conditions can also be rationally achieved by introducing active non-covalent intermolecular interactions in the matrices^45,46^. For instance, a recent study has demonstrated this concept by relying on the strong halogen and hydrogen bonding between an amorphous polymer matrix and the embedded organic luminophore to effectively minimize vibrational dissipation and enable bright phosphorescence (Fig. 3k–l)^45^. In this work, an organic luminophore 2,2’-(2-bromo-5-formyl-1,4-phenylene)bis(oxy)diacetic acid (**G1**) with a bromoaldehyde core and carboxylic acid side chains was rationally embedded within amorphous PVA with hydrogen bonds (Fig. 3k). In principle, the bromoaldehyde core of **G1** was expected to induce strong intermolecular halogen bonds between luminophores to suppress their vibrational dissipation and improve ISC (Fig. 3l). In addition, the carboxylic acid side chains of **G1** would facilitate the formation of strong intermolecular hydrogen bonds between **G1** and PVA to strengthen the restriction of luminophore vibrational loss. Additionally, the PVA–PVA hydrogen bonding would minimize diffusion of the polymer matrices. These intermolecular interactions synergistically reinforced RTP of **G1** via the strengthened restriction of vibrational and diffusional motions. This was clear from the strong RTP exhibited by the **G1**-PVA hybrid system, where its high phosphorescence quantum efficiency of 24% was approximately three times higher than that of **Br6A**-iPMMA.

**Fig. 4:**
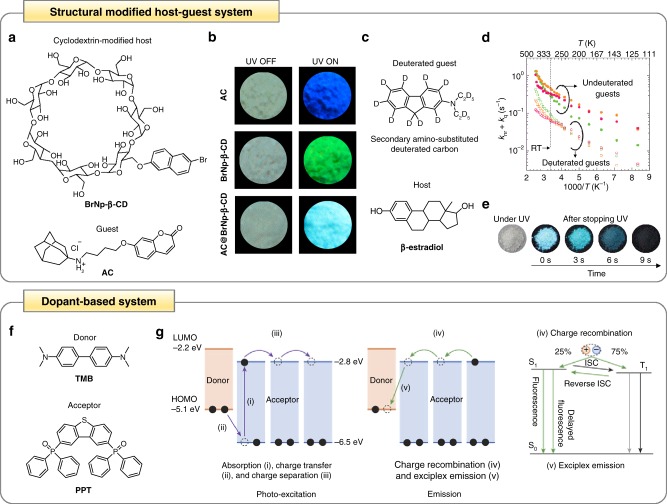
Enhancement of organic RTP based on structural modified host–guest system and dopant system. **a** Chemical structures of the cyclodextrin-modified host **BrNp-β-CD** and the guest **AC**. **b** Photographs of different states of the individual guest and host, as well as the host–guest system under daylight (left) and UV light excitation (right). **c** Chemical structures of the secondary amino-substituted deuterated guest and the host β-estradiol. **d** Sum of the non-radiative triplet deactivation rate (*k*_nr_) and triplet quenching rate (*k*_q_) of a range of host–guest films in which the host was β-estradiol and the secondary amino-substituted aromatic hydrocarbon guests were either deuterated (open circles) or not (solid circles). **e** Photographs of different states of the deuterated host–guest system under UV light excitation and after switching it off over time. **f** Chemical structures of the donor (guest) **TMB** and the acceptor (host) **PPT**. **g** Schematic illustration of the mechanism of RTP enhancement based on dopant system where charge-transfer states are formed during photoexcitation (i and ii), followed by the formation of charge-separation states (iii) and the eventual exciplex emission (iv and v). **a** and **b** are adapted with permission from ref. ^47^. Copyright (2018) American Chemical Society. **c**, **d**, and **e** are adapted with permission from ref. ^48^. Copyright (2013) Wiley-VCH Verlag GmbH & Co. **f** and **g** are reproduced with permission from ref. ^49^. Copyright (2017) Springer Nature

Besides via rigid matrix host–guest systems, the RTP of amorphous organic luminophores has been shown to be significantly enhanced via a structural modified host–guest system (Fig. 4a–e)^47,48^. To illustrate this enhancement principle, a rigid steroidal compound β-cyclodextrin (β-CD) was recently modified with various phosphorescent moieties to prepare a series of non-crystalline metal-free single RTP luminophores, including 6-bromo-2-naphthol-modified β-cyclodextrin (**BrNp-β-CD**) (Fig. 4a)^47^. These amorphous small molecular CD derivatives exhibited intense RTP luminescence in solid-state with phosphorescence lifetimes between 0.96 and 2.67 ms. This phenomenon was ascribed to the strong intermolecular hydrogen bonding between adjacent CDs, which mitigated the non-radiative vibrational deactivation of luminophores and protected them from quenchers, leading to efficient RTP emission. β-CD generally has a cavity, which is known to be able to host a plethora of guest molecules for the generation of a host–guest complex. As a proof of concept, a water-soluble fluorescent coumarin derivative (1s,3s)-N-(4-((2-oxo-2H-chromen-7-yl)oxy)butyl)adamantan-1-aminium chloride (**AC**) was introduced as a guest molecule into the cavity of **BrNp-β-CD** host to generate a structural modified host–guest complex **AC@BrNp-β-CD** (Fig. 4b). The supramolecular system possessed dual-emission properties originated from the individual components of **AC** and **BrNp-β-CD** and was capable of emitting multicolor luminescence, including white-light emission when excited at 295 nm. Intriguingly, this emission color could be manipulated by tuning the molar ratio of **AC** and **BrNp-β-CD**, or the excitation wavelength.

In addition to host molecules, modification can also be applied to guest molecules to reinforce the overall RTP performance. One of the earliest studies in this area formulated a structural modified host–guest system based on deuterated guest molecules (Fig. 4c–e)^48^. Specifically, the unique metal-free organic host–guest RTP systems were achieved through synergistic integration of a secondary amino-substituted deuterated carbon as a phosphorescent guest with its hydroxyl steroid analog as a host matrix (Fig. 4c). The two individual components of this amorphous host–guest system played crucial roles in affording long-lived RTP emission. In particular, the different amorphous hydroxyl steroidal compounds, such as β-estradiol, used as hosts possessed high rigidity and oxygen barrier characteristics, which could heavily suppress the quenching of long-lived triplet excitons. On the other hand, the aromatic hydrocarbons employed as guests were highly deuterated and secondary amino-substituted to minimize their non-radiative relaxations and enhance ISC. Interestingly, the advantage of highly deuterated guests were particularly pronounced in one of the characterization studies with β-estradiol as the host matrix (Fig. 4d). Various deuterated secondary amino-substituted aromatic hydrocarbon guests and their undeuterated counterparts were synthesized and combined with β-estradiol to generate a series of structural modified host–guest systems. Over a range of temperatures from 111 to 500 K, deuteration of the aromatic hydrocarbons was noted to reduce both the non-radiative triplet deactivation rate (*k*_nr_) and triplet quenching rate (*k*_q_), which is crucial to achieve long-lived triplet excitons and effective phosphorescence emission. Crucially, by leveraging on this RTP enhancement concept, persistent phosphorescence with a lifetime of >1 s and a quantum yield of >10% could be realized for a pure organic amorphous host–guest complex under ambient conditions (Fig. 4e).

One of the most exciting breakthroughs in the enhancement of RTP of pure organic luminophores has been reported most recently, in which organic long persistent luminescence was realized based on a dopant-based host–guest system (Fig. 4f, g)^49^. This work was largely inspired by the common use of mixtures of electron-donating and -accepting molecules to generate charge-separated states in organic photovoltaics^82,83^. Leveraging on a simple mixture of two organic molecules, i.e., *N,N,N’,N’*-tetramethylbenzidine (**TMB**) and 2,8-bis(diphenylphosphoryl)dibenzo[b,d]thiophene (**PPT**) serving as electron-donating and electron-accepting molecules, respectively, **TMB**/**PPT** amorphous film with a remarkable phosphorescence lifetime of >1 h was demonstrated (Fig. 4f). The unique features of this long lifetime luminescence enhancement principle lie in the generation of long-lived intermediate charge-separated states and their gradual recombination, which proceeded in sequential steps (Fig. 4g (i–v)). Firstly, upon photoexcitation, the electrons from the highest occupied molecular orbital of **TMB** transferred to that of **PPT**, resulting in the creation of intermediate charge-transfer states between **PPT** and **TMB** (Fig. 4g (i) and Fig. 4g (ii)). Next, through charge-hopping among the **PPT** molecules, the **PPT** radical anions would diffuse to sequester the **TMB** radical cations, leading to the formation of stable charge-separated states (Fig. 4g (iii)). The generation of these intermediate persistent charge-separated states was essential to retard charge recombination in order to realize long-lived emission. The subsequent charge recombination of the **TMB** radical cations and **PPT** radical anions (Fig. 4g (iv)) would then produce exciplexes (i.e., 25% singlet exciplexes and 75% triplet exciplexes (Fig. 4g (v)). As the photogenerated radical anions and cations could maintain their energy and accumulate in the **TMB/PPT** blend, the exciplex emission could last for an extended period of time after the removal of photoexcitation. Interestingly, reverse ISC through thermal activation could occur in this exciplex system due to the existence of a small energy gap between S_1_ and T_1_. It is important to highlight that the duration of the persistent phosphorescence emission was strongly dependent on several factors, such as the excitation duration and power, sample temperature, and concentration of the **TMB** dopant. For instance, to have a long phosphorescence lifetime, a low **TMB** concentration was needed as this could ensure a large distance between **TMB** and **PPT**, which in turn, maintained a low recombination probability of **PPT** radical anions with **TMB** radical cations.

Overall, the keys to achieving long-lived and bright RTP emission lie in the enhancement of ISC process coupled with the heavy suppression of non-radiative deactivation pathways of triplets. Therefore, by satisfying these crucial requirements, the functions and performance of pure organic RTP luminophores can be maximized for different applications through rational design and enhancement strategies.

## Advanced processing of pure organic RTP luminophores for biological applications

Despite the fact that organic luminophores with enhanced RTP have only started to be rationally designed in the last few years, active explorations have been channeled towards identifying their potential applications. These primarily include information encryption for anti-counterfeiting labeling, organic optoelectronics, ultrahigh density optical recording, and sensing^7,8^. Several key features that render organic RTP luminophores attractive for these applications, as compared to conventional short-lived organic fluorophores and thermally activated delayed fluorescent molecules, are their external stimuli-driven dynamic and reversible luminescence, and persistent room-temperature luminescent lifetime of beyond milliseconds to a few seconds. Moreover, it is possible to use pure organic RTP luminophores directly for these applications without the need for complex processing. As in-depth discussions on the potential applications of organic RTP luminophores in these areas have been previously covered in several reviews^7,8^, we therefore focus more on the emerging biological applications of pure organic RTP luminophores.

Organic RTP luminophores typically need to be specifically formulated for biological applications. This is because the effective utilizations of organic RTP luminophores for biological sensing and imaging hinge on stringent requirements, such as long-wavelength and bright RTP emission, suitable size, good water dispersibility, and excellent biocompatibility. As a promising application of organic RTP, biological imaging benefits from the utilization of organic luminophores with long-wavelength luminescence. Because of this, various organic RTP luminophores with long-wavelength (e.g., red emission) have been increasingly developed through ingenious approaches. These include utilizing heteroaromatic molecules with red emission (e.g., benzo[2,1,3]thiadiazoles)^21^, expanding π–conjugated structures^34^, and introducing appropriate red emitter dopants to facilitate the exciplex-dopant energy transfer and hence, redshift the emission wavelength^84^.

Apart from specific strategies in designing pure organic RTP luminophores with long-wavelength emission, there is a crucial need to process these luminophores so that they can remain sufficiently emissive in biological environment. This is because, when organic RTP luminophores are in contact with oxygen in aqueous media, their triplet excited states can be easily quenched^48^. Encouragingly, recent years have seen the emergence of various strategies to address this issue. Of particular interests are nanocrystallization of amorphous phosphorescent aggregates^57^ and polymer-assisted nanoencapsulation of phosphorescent luminophores^37,58–60^.

### Nanocrystallization

Organic RTP luminophores are highly attractive for biological applications, such as phosphorescence and lifetime imaging. This is because, with long lifetimes extending beyond milliseconds to a few seconds, organic RTP luminophores have the capability to minimize biological tissue autofluorescence and background interference, which typically last up to several nanoseconds. At the same time, pure organic RTP luminophores are able to mitigate the extended utility of external illumination source and they possess excellent biocompatibility. Although organic RTP crystals possess such attractive features, there have been minimal successful attempts to use them for biological applications. This has been attributed to several factors. To date, a majority of the demonstrated bright pure organic RTP luminophores possess short-wavelength emissions. Many current strategies to yield bright organic RTP are not compatible with biological applications as additional molecules or matrices have been deliberately incorporated for the synthesis of these compounds. Unfortunately, the presence of these additives may interfere with biological systems and compromise the biocompatibility of the eventual organic RTP luminophores. Meanwhile, for those organic compounds exhibiting crystallization-induced phosphorescence, their sizes are typically too large for biological applications.

**Fig. 5:**
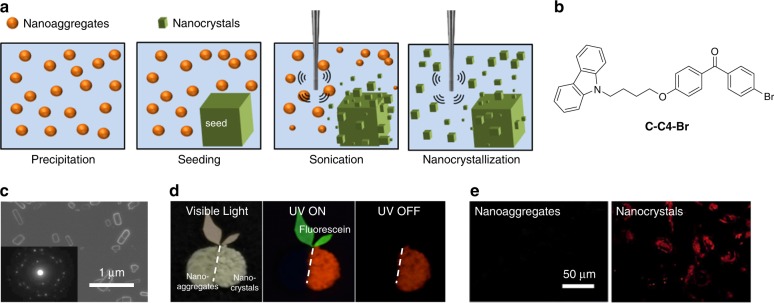
Nanocrystallization of amorphous nanoaggregates to yield organic RTP contrast agents for bioimaging. **a** Schematic illustration of the seed-assisted nanocrystallization process. **b** Chemical structure of **C-C4-Br** with red persistent RTP. **c** Scanning electron microscopy image of the **C-C4-Br** nanocrystals. Inset shows the selected area electron diffraction pattern of the nanocrystals. **d** Photoluminescence of the **C-C4-Br** amorphous nanoaggregates and nanocrystals (both in orange) and fluorescein (green) under various illumination states. **e** Confocal laser scanning microscopy images of breast cancer cells treated with amorphous nanoaggregates (left) and nanocrystals (right) of **C-C4-Br**. **a** is adapted with permission from ref. ^85^. Copyright (2017) Wiley-VCH Verlag GmbH & Co. **b**, **c**, **d**, and **e** are adapted with permission from ref. ^57^. Copyright (2017) Wiley-VCH Verlag GmbH & Co

To solve these problems, our group has recently developed a unique nanocrystallization strategy (Fig. 5)^57^. As shown in Fig. 5a, the nanocrystallization strategy relies on three primary processes, i.e., precipitation, seeding, and ultrasonication, to maximize the nucleation rate of crystals while simultaneously minimize their growth rate^85^. Amorphous nanoaggregates are initially formed through injection of an organic compound in a good solvent (e.g., tetrahydrofuran) into its antisolvent (e.g., water). Once crystal seeds of the organic compound are introduced to direct and induce crystallization, the suspension is subjected to ultrasonication to promote detachment of the crystals from their seeds, which serve as new seeds to accelerate the nucleation rate and formation of nanocrystals. Interestingly, the crystal size can be simply fine-tuned by varying the ratio of the antisolvent to good solvent. The conversion of amorphous nanoaggregates to nanocrystals is typically accompanied by changes in optical properties. The nanocrystallization method is widely applicable to a range of organic molecules with various chemical structures and sizes.

To realize nanocrystals with bright long-wavelength phosphorescence for biological applications, we designed a new RTP molecule (4-(4-(9*H*-carbazol-9-yl)butoxy)phenyl)(4-bromophenyl)methanone (**C-C4-Br**) by adding a butoxy spacer in between the carbazole and the 4-bromobenzophenone groups to spatially separate them (Fig. 5b). This soft spacer was incorporated in order to enhance the overall heavy halogen atom effect through strengthening the bond between the carbazolyl plane of one molecule and the bromine atom of the neighboring molecule. Following the steps shown in Fig. 5a, the nanocrystals of **C-C4-Br** with rod-like morphology and a size of 180 nm were synthesized (Fig. 5c). Along with the generation of **C-C4-Br** nanocrystals, their RTP emission was assessed (Fig. 5d). Of all three specimens under examination, i.e., **C-C4-Br** nanoaggregates, **C-C4-Br** nanocrystals, and fluorescein, only the nanocrystals were emissive and observable under all illumination conditions, in the presence and absence of visible light and UV light irradiations. Both the nanocrystals and nanoaggregates of **C-C4-Br** were then used for in vitro phosphorescence imaging of breast cancer cells (Fig. 5e). No photoluminescence could be detected from the cancer cells treated with the nanoaggregates. In contrast, the nanocrystal-treated cells displayed bright red phosphorescence emission. Furthermore, the phosphorescence lifetime of **C-C4-Br** nanocrystals was 0.14 s and could be utilized to clearly differentiate the nanocrystal-labeled cells from the fluorescein dye interference and background autofluorescence. The nanocrystals also displayed excellent biocompatibility. All these have clearly illustrated the effective cellular internalization and highly emissive phosphorescence of nanocrystals, and most importantly, the superiority of nanocrystalline RTP luminophores over their amorphous counterparts for biological applications.

### Nanoencapsulation

**Fig. 6:**
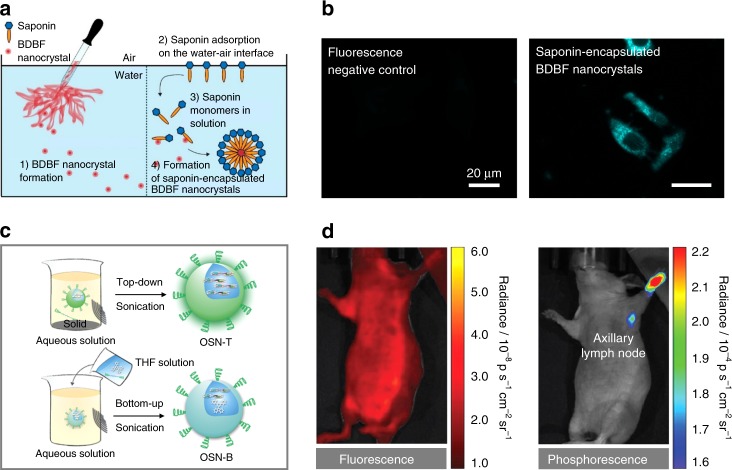
Nanoencapsulation and surface functionalization of nanocrystals and amorphous nanoaggregates to yield organic RTP contrast agents for bioapplications. **a** Schematic illustration of the nanoencapsulation of **BDBF** RTP nanocrystals using saponin. **b** Confocal laser scanning microscopy images of HeLa cells treated with pure **BDBF** nanocrystals (left) and saponin-encapsulated **BDBF** nanocrystals (right). **c** Schematic illustration of the syntheses of amorphous organic semiconducting nanoparticles with ultralong RTP, i.e., OSN-T and OSN-B, using top-down (top) and bottom-up (down) approaches, respectively. **d** Fluorescence (left) and ultralong phosphorescence (right) imaging of lymph node in living mice using intradermally administered OSN-T. **a** and **b** are adapted with permission from ref. ^58^. Copyright (2017) American Chemical Society. **c** and **d** are adapted with permission from ref. ^59^. Copyright (2017) Wiley-VCH Verlag GmbH & Co

Further to the top-down nanocrystallization, bottom-up approach such as nanoencapsulation was also used to process organic RTP luminophores for biological applications^37,58–60^ (Fig. 6). Using this approach, pure organic RTP luminophores are generally wrapped within an amphiphilic shell to endow the eventual structures with excellent water dispersibility and good cellular uptake. For example, pure organic RTP nanocrystals of **BDBF** (Fig. 2o) have recently been encapsulated within an amphiphilic saponin with a unique permeabilization property for improved intracellular delivery and imaging of live cancer cells (Fig. 6a, b)^58^. The saponin-encapsulated **BDBF** nanocrystals were prepared by introducing saponin into the **BDBF** nanocrystals dispersed in phosphate-buffered saline (Fig. 6a). These processed nanocrystals had a uniform size of about 460 nm. To demonstrate the advantage of the saponin-based encapsulation process, the saponin-encapsulated **BDBF** nanocrystals were used for in vitro phosphorescence imaging of HeLa cells. It was noted that these nanocrystals were able to diffuse through the permeabilized membrane of HeLa cells, stain the cells with high photostability, and maintain the persistent lifetimes of up to 100 ms, which is significantly longer than background autofluorescence (Fig. 6b).

Recent studies have also shown that the nanoencapsulation technique can be used to process pure organic RTP luminophores prepared from both top-down and bottom-up synthetic strategies (Fig. 6c, d)^59,60^. For instance, a series of organic RTP luminophores, including **DPhCzT** (OS1) (Fig. 2i), was prepared through both the top-down transformation of solid crystals into nanoparticles (OSNs-T) and the bottom-up nanoprecipitation of nanoparticles (OSNs-B)^59^. An amphiphilic triblock copolymer of PEG-*b*-PPG-*b*-PEG (F127) was subsequently utilized to encapsulate OSNs-T and OSNs-B in order to stabilize and endow them with good aqueous dispersibility (Fig. 6c). Although each pair of the nanoparticles possessed the same phosphorescence dye and spherical morphology, the hydrodynamic diameter of all OSNs-B (~20 nm) was notably smaller than that of OSNs-T (~70 to 80 nm). Both sets of organic nanoparticles did not induce any noticeable cytotoxicity. Interestingly, all three OSNs-T exhibited enhanced phosphorescence as compared to their OSNs-B counterparts, possibly due to the stronger molecular packing and enhanced stabilization of triplet excitons. In addition, of all OSNs-T, OSN1-T, which was prepared from **DPhCzT**, exhibited the strongest and longest phosphorescence, which could be detected using a whole-animal imaging set-up after switching off external illumination. OSN1-T was then selected and used for proof-of-concept in vivo long-term phosphorescence imaging (Fig. 6d). Even at a low nanoparticle concentration of 7.5 nM, the phosphorescence of OSN1-T could be clearly observed in the subcutaneous tissue of living mice. Importantly, due to the mitigation of tissue autofluorescence, OSN1-T enabled a highly sensitive imaging of axillary lymph node in living mice with a signal-to-noise ratio of 40, while fluorescence imaging could not distinguish the lymph node from normal tissue.

Separately, the F127-mediated nanoencapsulation strategy was also used to wrap organic RTP aggregates **CPhCz** (Fig. 2m) to generate water-dispersible RTP nanoparticles for in vitro phosphorescence imaging^37^. These nanoparticles had an average size of about 68 nm and exhibited a long phosphorescence with an emission lifetime of ~0.65 s. The attractive RTP feature of the nanoencapsulated **CPhCz** nanoparticles was then utilized for labeling and phosphorescence imaging of hepatocellular cells. Similar approach to encapsulate organic long-lived RTP luminophores, i.e., specific derivative of 10-phenyl-10*H*-phenothiazine-5,5-dioxide, using F127 to generate water-dispersible nanoparticles for in vivo phosphorescence imaging has also been demonstrated in a recent study^60^. The F127-encapsulated nanoparticles were intradermally administered into living mice and their ultralong RTP was clearly observed.

In short, these studies have demonstrated general applicability of nanoencapsulation method in generating dispersible RTP nanoparticles with retained phosphorescence brightness and lifetimes for real-time phosphorescence imaging.

## Summary and outlook

Motivated by the rapid advancements in the molecular design, formulation, and processing strategies, pure organic long-lived RTP luminophores have been increasingly demonstrated in recent years. Efficient singlet-triplet ISC, slow triplet excited state decay rate, and suppressed non-radiative relaxation rates, which are essential factors to the realization of pure organic RTP, have been enabled through various molecular design strategies, such as halogen bonding^33–35^, H-aggregation^36–38^, and *n*–π transition^39,40^. Concurrently, the emergence and development of various organic RTP enhancement approaches, notably co-crystallization^34,41–43^, rigid matrix host–guest system^44–46^, structural modified host–guest system^47,48^, and dopant-based system^49^, have significantly enhanced and elevated the performance of pure organic RTP luminophores, in terms of phosphorescence quantum efficiency and lifetime.

As an emerging class of luminescent materials, pure organic luminophores with persistent and bright RTP have remarkable properties, such as configurable molecular structures, tunable photoluminescence properties, long phosphorescence emission lifetime, and stimuli-responsiveness. Because of these attractive properties, they are poised to have tremendous potential applications. For instance, the long-lived phosphorescence of organic RTP luminophores coupled with their stimuli-responsiveness have been actively explored for applications in sensing and anti-counterfeiting labeling for data security protection. Additionally, the extended luminescence lifetimes of organic RTP luminophores are beneficial to the mitigation of background autofluorescence, which enables highly specific and sensitive biological imaging. With such unique features and potential applications, the value and importance of pure organic RTP luminophores have grown exponentially in the last several years, spurring increasing explorations to uncover all aspects of this unique group of organic materials. Some of these important aspects include: (1) re-investigations and deeper elucidations of the fundamental mechanisms of a range of pure organic RTP luminophores (i.e., organic small molecules, macromolecules, and carbon dots), (2) more facile and efficient design of novel and robust pure organic RTP luminophores with high brightness and long lifetime, (3) further enhancements of the phosphorescence features and performance of these organic RTP luminophores (e.g., organic RTP lifetime, long-wavelength emission, full-color emission, etc.), (4) comprehensive evaluations of the biological effects of pure organic RTP luminophores, and (5) further exploitations of the potential applications of pure organic RTP luminophores.

Firstly, while increasing studies have highlighted the uniqueness and superiority of pure organic RTP luminophores, it is important to highlight that the development of this field is still in its infancy. As a result, certain proposed mechanisms underlying the occurrence of organic RTP may warrant deeper investigations or revisits. For instance, although a large body of literature has demonstrated the strong persistent RTP emission of the carbazole-based organic compounds when they exist in crystal state, there has been a correction report pointing that this RTP emission could possibly be originated from small amount of impurities^76^. This interesting observation has underlined the importance of sample purity before any phosphorescence characterization. As such, some of the currently well-received principles may require further verifications. More efforts are definitely needed to elucidate these RTP enhancement mechanisms thoroughly before the rational design strategies can be tested and established.

Despite the fact that a large number of investigations to date have focused primarily on RTP mechanisms of organic small molecules, those of other structures, such as carbon dots^61,62^ and macromolecules (e.g., proteins, starch, carbohydrates, cellulose, and conjugated polymers)^63,64^, have not been widely explored. For example, macromolecules from natural products have been reported to exhibit obvious long-lived phosphorescence under ambient conditions^63^, although the driving mechanisms are still not well understood and await further exploration. Similarly, preliminary studies on carbon dots have demonstrated that these nanomaterials are capable of emitting long-lived phosphorescence under ambient conditions when they are embedded in rigid matrices, such as PVA^86^ and silica gel^87^. More efforts are consequently essential to unravel the mechanisms, rational design, and enhancement strategies of the long-lived RTP phenomenon of more complex organic materials.

Secondly, leveraging on more comprehensive insights into the mechanisms of a wide range of pure organic RTP luminophores, increasing focus should be placed on developing more facile and efficient design strategies to realize highly robust organic RTP luminophores. Currently, there are still limited types of pure organic RTP luminophores with high brightness and persistent RTP emission. Although this may be partly attributed to the infancy of the field, it is worth mentioning that most of the organic RTP luminophores have been designed through complex approaches. Moreover, these luminophores can only manifest their RTP emission under stringent conditions. All these have inevitably limited the types of available high-performance organic RTP luminophores for practical applications. An increased understanding of pure organic RTP is, therefore, crucial to overcome this limitation as the newly gained insights will be beneficial to the development of a more rational and facile design framework to expand the library of pure organic RTP luminophores.

Thirdly, in addition to a deeper understanding of the fundamental mechanisms and improved designs of pure organic RTP luminophores, significant enhancements to their phosphorescence performance should be carried out in tandem. It is noteworthy that the phosphorescence features of organic RTP luminophores are still not comparable to those of their inorganic counterparts, especially in terms of phosphorescence quantum efficiency and lifetime. Encouragingly, there are clear signs that recent activities have been geared towards bridging the performance gaps between organic and inorganic RTP luminophores. This is evident from one of the most recent demonstrations of a persistent organic dopant-based RTP system with a remarkably long phosphorescence lifetime of ~1 h under ambient conditions^49^. With this breakthrough, there has been growing confidence that maximization of the phosphorescence performance of pure organic RTP luminophores to rival that of inorganic luminophores could possibly be achieved in the near future. Systematic investigations are thus needed for pushing this performance limit of organic RTP luminophores.

Fourthly, another important aspect of pure organic RTP that requires deeper examinations is the biological effects of organic RTP luminophores. While there has been great excitement for the imminent utilization of organic RTP luminophores for a wide range of applications, further advancement needs to proceed with care. In fact, to fully realize the practical applications of pure organic RTP luminophores, specifically their biological applications, it is imperative to understand their biological characteristics so that these functional nanomaterials can be rationally engineered and used safely. Unfortunately, to date, the information on the toxicological profiles and biocompatibility of pure organic RTP luminophores is still relatively scarce and largely unknown. With the increasing explorations into potential applications of pure organic RTP luminophores, their biological characteristics definitely deserve more attention and investigation.

Lastly, more potential applications of organic RTP luminophores need to be further exploited and developed. In fact, it is essential to identify the “killer” applications of pure organic RTP luminophores, which they are capable of revolutionizing and which they will have competitive advantages over conventional and other similar nanomaterials. Additionally, while many studies have reported the promising utilizations of pure organic RTP luminophores, most of these demonstrations have largely been confined at an early proof-of-concept stage. More works are required in tandem to move these early-stage demonstrations into maturation and possible commercialization.

In summary, it remains to be seen if organic RTP luminophores can rival or even surpass their inorganic counterparts and live up to the expectations. However, with a more in-depth understanding of the fundamental mechanisms of the phenomenon of organic RTP coupled with the further development in the rational design and RTP enhancement strategies, we envision that pure organic luminophores with long-lived and bright RTP emission may find potential widespread applications in the near future.
